# Frontline health workers as brokers: provider perceptions, experiences and mitigating strategies to improve access to essential medicines in South Africa

**DOI:** 10.1186/s12913-014-0520-6

**Published:** 2014-11-05

**Authors:** Bvudzai Priscilla Magadzire, Ashwin Budden, Kim Ward, Roger Jeffery, David Sanders

**Affiliations:** University of the Western Cape, School of Public Health, Private Bag X17, Bellville, 7535 South Africa; University of Basel, Swiss Tropical and Public Health Institute, Basel, Switzerland; University of the Western Cape, School of Pharmacy, Bellville, South Africa; University of Edinburgh, School of Social and Political Science, Edinburgh, Scotland

**Keywords:** Access to medicines, Provider perceptions, Health services, Broker, Empathy, HIV, Diabetes, Depression, TB, South Africa

## Abstract

**Background:**

Front-line health providers have a unique role as brokers (patient advocates) between the health system and patients in ensuring access to medicines (ATM). ATM is a fundamental component of health systems. This paper examines in a South African context supply- and demand- ATM barriers from the provider perspective using a five dimensional framework: *availability* (fit between existing resources and clients’ needs); *accessibility* (fit between physical location of healthcare and location of clients); a*ccommodation* (fit between the organisation of services and clients’ practical circumstances); *acceptability* (fit between clients’ and providers’ mutual expectations and appropriateness of care) and *affordability* (fit between cost of care and ability to pay).

**Methods:**

This cross-sectional, qualitative study uses semi-structured interviews with nurses, pharmacy personnel and doctors. Thirty-six providers were purposively recruited from six public sector Community Health Centres in two districts in the Eastern Cape Province representing both rural and urban settings. Content analysis combined structured coding and grounded theory approaches. Finally, the five dimensional framework was applied to illustrate the interconnected facets of the issue.

**Results:**

Factors perceived to affect ATM were identified. Availability of medicines was hampered by logistical bottlenecks in the medicines supply chain; poor public transport networks affected accessibility. Organization of disease programmes meshed poorly with the needs of patients with comorbidities and circular migrants who move between provinces searching for economic opportunities, proximity to services such as social grants and shopping centres influenced where patients obtain medicines. Acceptability was affected by, for example, HIV related stigma leading patients to seek distant services. Travel costs exacerbated by the interplay of several ATM barriers influenced affordability. Providers play a brokerage role by adopting flexible prescribing and dispensing for ‘stable’ patients and aligning clinic and social grant appointments to minimise clients’ routine costs. Occasionally they reported assisting patients with transport money.

**Conclusion:**

All five ATM barriers are important and they interact in complex ways. Context-sensitive responses which minimise treatment interruption are needed. While broad-based changes encompassing all disease programmes to improve ATM are needed, a beginning could be to assess the appropriateness, feasibility and sustainability of existing brokerage mechanisms.

## Background

South Africa is in the midst of a profound health transition that is characterised by a multiple burden of disease that includes communicable and non-communicable diseases [1]. This has increased demand for essential medicines in the public health system which caters for almost 70% of the country’s population [2]. The South African health system is plagued by a human resources crisis and the primary health care system is a mainly nurse-driven service [3]. Nurses together with other frontline health providers (hereinafter referred to as providers) i.e. pharmacy personnel (pharmacist or pharmacist assistant) and doctors where present, play a strategic and key role as brokers (usually as patient advocates) between patients and the health system at the point of care. Thus, understanding their perspectives and actions can strengthen planning and provision of future services [4]. We draw on the work of Long et al. in analysing the role of brokers and their capacity to link disparate groups in the provision of health care [5].

We explore the brokerage aspect in ATM, a fundamental component of health systems. Although no universal definition of access exists [6], for our purposes, we adopt a definition which describes access as the fit between supply and demand of healthcare along five dimensions: *availability* (fit between existing resources and clients’ needs); *accessibility* (fit between physical location of healthcare and location of clients); a*ccommodation* (fit between the organisation of services and clients’ practical circumstances); *acceptability* (fit between clients’ and providers’ mutual expectations and appropriateness of care) and *affordability* (fit between cost of care and ability to pay) [7].

Many studies in South Africa highlight supply- and demand-side barriers to ATM such as treatment-related costs [8-10], waiting times at health sites [11], poor provider-patient interaction [12,13], and structural inequities, particularly among rural populations [14]. Most of these findings derive from patient reports. In this study, however, we explore provider perspectives, within the same framework commonly utilised to understand patient perspectives, to ascertain their perceptions of these barriers to accessing essential medicines, i.e. those medicines that satisfy the priority health care needs of the population [15]. The literature on providers and their interactions with patients tends to cast them in a negative light [16-19]. However, little attempt has been made to investigate how providers view and attempt to mitigate the daily challenges they encounter in patient care. The objective of this paper is to address this gap by highlighting the views of primary health care providers on supply- and demand-related ATM barriers in the Eastern Cape Province. A second objective is to highlight the ad hoc, empathic responses that providers in their broker capacity employ in their day-to-day practise to mitigate challenges faced by patients.

## Methods

### Study setting

The Eastern Cape Province has an estimated population of 6.8 million, contributing about 13.5% of the country’s population [20]. 62% of its population is rural and highly deprived, contributing significantly to poor health outcomes [14]. At the time of the study, primary level facilities were managed by two local authorities, the Provincial Department of Health (responsible for the majority of sites) and municipalities in urban areas. However our sample consisted of only the Provincial Department of Health sites.

### Design

This research is a component of a larger research project on Accessing Medicines in Africa and South Asia (AMASA), which investigated supply and access to essential medicines for TB, HIV, Type-2 diabetes and unipolar depression. The present cross-sectional, qualitative study is based on key informant interviews with nurses (health centre managers also commonly known as facility managers and disease programme managers), doctors and pharmacy personnel (pharmacists or pharmacist assistants or dispensing nurses). Thirty-six providers were purposively recruited from six public sector Community Health Centres (CHCs) in two districts (one rural and one urban) in the Eastern Cape Province. CHCs were selected because they are the first level of care in the health system and provide a broad range of essential medicines and primary health care services in the public system. Recruitment of respondents from rural and urban settings aimed to consider varying conditions of access in different geographic locations. Four of the sites were rural and two were urban. Key informant interviews addressed themes regarding access to selected essential medicines which are available in the public sector: lamivudine (3-TC), part of the first line anti-retroviral therapy (ART) for adults infected by Human Immunodeficiency Virus (HIV); rifampicin, an antimycobaterial drug for tuberculosis (TB); metformin, a blood glucose lowering drug for type 2 diabetes; and two antidepressants, fluoxetine and amitriptyline. At each CHC, the facility manager, pharmacy personnel and one or more representatives per essential medicine (nurse and/or doctor) were recruited. Unlike in towns where providers manage a single disease programme, in rural areas, they manage multiple disease programmes because of human resource shortages. Sufficiency of sample size was determined by saturation, i.e., when the collection of new data did not shed any further light on the issue under investigation.

Interviews were conducted during the months of March and April of 2012. This study protocol was approved by the University of the Western Cape Ethics Committee and both the National and Provincial Department of Health Committees. Each participant gave both verbal and written consent to participate in the research and was given information leaflets with a brief description of the study and contact details of the responsible institution and contact person.

### Instruments

Key informant interviews with providers were conducted using a semi-structured interview guide. Interview guides for each stakeholder group contained a set of broader themes with corresponding questions and probes as informed by the study objectives. However, the common lines of enquiry among all stakeholder groups that were relevant for this paper focussed on identifying the most significant health system and patient level barriers to accessing essential medicines and how these differ or are similar across settings (rural and urban); type of medicine (or disease condition); and the strategies that exist within the health system to overcome these barriers, including the availability of alternative medicines access points such as mobile clinics. Key informant interviews were digitally recorded and transcribed.

### Data analysis

The transcripts were imported to a computer programme, MAXQDA version 10 for organisation and content analysis. Content analysis is a standard approach to analysing qualitative data to identify patterns, themes and biases in that material [21]. The analysis applied structured coding and grounded theory approaches to the narratives. Coded segments of transcripts were then retrieved and further analysed for recurrent or conflicting response patterns. Finally, coded segments were categorised according to the five dimensional framework. The coding frameworks were discussed with the authorship team at both stages.

## Results

The sample and background characteristics of respondents are shown in Table 1 below.

**Table 1:** **Characteristics of respondents N(36)**

Antiretroviral Therapy (ART) programme nurses	**7**
Tuberculosis (TB) programme nurses	6
Chronic care nurses (responsible for management of diabetes and/or mental health programmes)	8
Doctors	3
Pharmacist/pharmacist assistant/dispensing nurses	6
Facility/health centre managers (nurses)	6
**Sex**	
Male	6
Female	30
Age (years)	Range: 24-63
	Median: 52
**Number of years working at the facility**	Range: <1-29
	Median: 8
**Number of years working in the health profession**	Range: <1-30
	Median: 26

Various supply- and demand-side barriers to essential medicine access were identified.

Providers also discussed the ad hoc strategies that they employ to address these challenges. Notably, although the investigation aimed to consider essential medicines for HIV, TB, diabetes and depression, we found minimal prescribing of antidepressants in this context. When prescribed, it is usually on a short term basis. However, we still included the findings with regard to antidepressants in the analysis. Below we elaborate provider perspectives with reference to the five dimensional framework and their mitigating strategies and recommendations.

### Availability

Stock outs (i.e. when health centres run out of medicines) were viewed, as a major challenge particularly in rural clinics. Providers in rural CHCs highlighted the lack of dedicated vehicles to transport medicines from CHCs that act as temporary storage for rural clinics as a hindrance to efficient distribution. Each of the four rural CHCs visited provided temporary medicine storage for more than 20 peripheral clinics located in the deep rural areas. However, at the time of the research there were no dedicated vehicles for transportation of medicines from the CHCs to the clinics. Vehicles allocated for other tasks were also expected to deliver medicines. In the past, medicines delivery was outsourced to a private company by the Provincial Department of Health, however, the contract with the service provider had expired (reportedly a couple of months before our research) and no other strategy had been put in place to address delivery of medicines more sustainably. As one health centre manager explained,

“*There is no challenge getting the drugs from the depot to here, the challenge is getting the drugs from here to the clinics because we have shortage of transport. All our vans are attached to programmes. They are used by mobile clinics, school health, circumcision so when there is a need for the clinics to get some drugs we have to borrow a van from that person. And the clinics are so many. We are having 18 clinics at the moment and they will soon be 22. We are short of vehicles because even the clinic supervisors don’t have a dedicated vehicle. The one that they are using, we used to take it when there is gas, it’s the only long base van so when we have to deliver gas to the clinics we need that van and when we need to deliver medication then you have to borrow”.***(Facility manager, CHC 1, rural)**

In the event of stock outs, providers reported that they either referred patients to alternative facilities or gave them another appointment date to return to the facility. Erratic supplies were reportedly one of the main factors leading to patients’ loss of confidence in local clinics. At one of the urban CHCs, it was reported that pharmacy personnel had conducted a study to determine the amount of money spent on travel to facilities and the number of taxis taken per trip. Their investigation found that 60% of patients on ART lived outside the catchment area and generally took up to two taxis to reach the CHC. Among the reasons for not attending the nearest clinic, stock outs and perceived quality of service ranked highly. Accounts from TB and ART programme nurses corroborate this finding:

*“We refer them to the local clinics but they come back because there is a shortage of treatment in the local clinics…we cannot chase them away because we know they need to take this treatment”.***(TB nurse, CHC6, urban)**

*“We have got so many facilities around here and they (patients) don’t want to go to those clinics even though they are only walking distance…Sometimes they say there are no drugs there…we are still trying to investigate because we are always told that drugs are being taken to the clinic, how come the patients say there are no drugs?”***(ART nurse, CHC 2, rural)**

### Accessibility

Key informants agreed that public transport to peripheral clinics, which public sector patients rely on heavily, is either absent or limited. According to providers, the district boundaries in rural locations, in particular, are out of sync with public transport networks. As a result patients opt for CHCs in town which are more accessible and safer to visit. The following accounts illustrate this point:

*“There is a challenge because this CHC is very far from other clients. Now the problem is there are residential clinics (small, rural clinics) and the client will give the report that there is no transport from my area to the clinic so I only get the transport from my area to town so in the morning it’s better to come to town, whereas they don’t belong to this catchment area, they belong to residential clinics…in the morning transport is going to town, maybe a bus or a van and you will find that they will be short of money and they will come after their old age pension grant and miss their appointment. We are having that problem with clients far away from us but we do register them because we don’t know what to do. We do accept them with their problem we give them the return date three days before the treatment is finished so that there is a buffer in case they are late”.***(ART nurse, CHC1, rural)**

*“…we are not only serving this area, our so called catchment, they (patients) say to us it’s better to take a taxi to come here than to go to the rural clinics because in the morning, transport wise it’s not very easy and with other localities they don’t have transport. They are afraid of being raped, so they prefer taking a taxi for twenty rand (approximately two United States dollars at time of research) to come to East London, and mind that is dependent maybe on the mother to get the money to come here”.***(Chronic care nurse, CHC5, urban)**

Lack of transport is largely attributed to poor infrastructure and is the reason for transport operators avoiding certain routes. The following sentiments were captured regarding difficulties for patients in the Transkei region, a former homeland and a deeply deprived area in the province:

*“… our roads are still not tarred, so some patients have to travel more than 50 km. You know 50 km in the rural areas is a lot, they can even take up to two hours because of the gravel roads…and in the rural areas by 5 p.m. the transport gets finished, so these people won’t have transport to go back home. Some of the patients have to wake up at 3 a.m. when they come to visit the clinic, like for reviews…it’s not fair”.***(Chief medical officer, CHC3, rural)**

### Accommodation

The down referral program which transfers patients to facilities nearest to their homes was implemented as a national strategy to improve ATM. There were mixed opinions about the success of down referral but there was consensus that uptake of services at the lower levels of care could increase if the quality of services, including the availability of medicines improved. Here accommodation of patient perceptions play a key role in the uptake of the intervention as illustrated below:

“*People want to go to a place where there is a doctor, where there is availability of medicines. Even if they are not going to be seen by that doctor…as long as they know that in the event that a condition warrants you to be seen by the doctor, the doctor will be there. There are people who don’t want to go to clinics on that basis; they know there are nurses only”.***(Pharmacist, CHC6, urban)**

*“…with the municipal clinics for example, they don’t have a doctor every day, they don’t have an x-ray machine, and they have certain days for immunization, for minor ailments, for chronics for example. Now here…you can come any day for immunization, you can come any time…and I think that is one of the reasons why they flock here. We’ve got many doctors, we’ve got six permanent doctors…they want a doctor, they want an x-ray, they want to come whenever they want to come”.***(TB nurse, CHC5, urban)**

*“We do transfer them (patients) to the local nearest clinic, but sometimes they don’t want to go there, they prefer to come here, they don’t get the treatment at the nearest clinic, they say the treatment was out of stock so they decided to come here, but we are trying to take (refer) them back to the nearest clinic so they can afford money-wise”.***(Facility manager, CHC1, rural)**

*“We are doing down referrals to the near sites but it’s not proper because patients are going there in those clinics and they say they didn’t get their medication”.***(ART nurse, CHC4, rural)**

In the context of working with the poor, proximity of health services to social services emerged as important because social grants are central to poor people’s livelihoods. Many informants associated patients’ choice of a health care facility with its closeness to other services such as social grant collection points and shopping centres. Providers reported that many patients rely on their or a family member’s social grant to cover transport expenses. This was confirmed by the research team when a particular facility waiting area was relatively empty on the day of grant collection compared to other days. The ability to combine medicine collection appointments with other personal business made it more appealing as indicated by the following accounts:

“*There are some clinics in other rural areas, but they say this is their town, Home Affairs is here, Social Development is here, Shoprite* [supermarket] *is here, so they say they will rather come to town, so they do everything and then come to the clinic”.***(TB nurse, CHC4, rural)**

“*We have got eleven communities that we are serving. They are very far from here, it’s more than 30 km…they are rural and they are struggling to come here, others are not getting any grant and they don’t have any money to come”.***(Chronic care nurse, CHC4, rural)**

Another important aspect in the organisation of disease control programmes is their inability to recognise that circular migration is a key feature among patients. Circular migrants are people who move between their places of origin to nearby urban centres or to other provinces in search of economic opportunities or for social reasons. For example, many people from the Eastern Cape Province move to the Western Cape Province seeking for economic opportunities and they travel between those provinces regularly. The provinces in some cases use different treatment protocols and this confuses not only the patients, most of whom recognise their medication by its physical appearance rather than generic names, but also providers, who feel overwhelmed by such scenarios as indicated by two nurses in rural settings.

*“In the Western Cape they have their own regimen, which is not the same as ours here because even for transfers, we always have that problem because we many not know that regimen. You will find that the drug that is in our regimen two, they are using it for regimen one. Now that is confusing”.***(TB nurse, CHC2, rural)**

*“Our concern is for people coming from KZN (Kwa-Zulu Natal province) who are on ATRIPLA (fixed-dose ART), that is one tablet and we are still using three separate drugs”.***(ART nurse, CHC1, rural)**

In some cases, patients travel without referral letters and as a result default on their treatment because they are apprehensive about going to the health centre without a referral letter. They delay seeking medical attention until they become seriously ill. The following narratives echo this point.

“*With diabetes and hypertension, patients will say I was in Cape Town to see my sick child, and we will ask them but why didn’t you carry your own clinic card, what about your own health?”***(Chief medical officer, CHC3 rural)**

*“…we tell them if you have to go somewhere come and report so that we provide extra medicine supply and if you are staying for a longer period then take the transfer letter. They don’t come and report and they don’t take their transfer letters. When they reach KZN, they are expecting the client to have the transfer letter so that they know exactly what is going on with him or her. Even if you trace by phone she says oh I am taking the medication here. How? Because you didn’t come to collect your medication and your transfer letter but they say no they are giving me. But then when she comes in two months later the card is still with that last date when she came here”.***(TB nurse, CHC2, rural)**

*“…they will tell you they have been to Cape Town but they don’t come with a card from Cape Town so we don’t know if they have taken the treatment or not”.***(Chronic care nurse, CHC3 rural)**

*“The patients are moving up and down that is why they are not adhering”.***(ART nurse, CHC2, rural)**

### Acceptability

Socio-cultural influences were also highlighted as fuelling stigma and the reason for some ART patients choosing to go to facilities that are further away from their homes, rather than nearer facilities. This was common among HIV patients and was not reported for other conditions. These three illustrations highlight this situation:

*“…they (ART patients) are not happy about being seen taking HIV treatment in their clinics…if one persists, you observe and if you find out that they might end up not taking the treatment, we admit that one”.***(Facility manager, CHC4, rural)**

*“They stigmatise this condition (HIV) and we need to educate the people. I have got a brother who is HIV positive. I didn’t know he was HIV positive until today…he has been getting treatment from an NGO in town. Now that NGO has closed because of financial issues, now they are referred to the hospital. I don’t know whether he is running away from me or the centre itself”.***(Facility manager, CHC6, urban)**

*“We have a patient that comes once a month; she’s an HIV positive lady and had TB also. She comes here … for her treatment. So patients will go where they want to go even if you made hundred packs to leave at the (local) clinic, they won’t go there. She spends five hundred rand (approximately 56 United States dollars at the time of research) to come here when it’s time for her checkup. When it’s not her checkup, her son comes but because she had TB she had to come two weekly you know but with the ARV, her son collects her ARVs. ARV’s are expiring in other clinics because they don’t want to go to there… That’s why we have an overflow of patients here”.***(TB nurse, CHC5, urban)**

### Affordability

Although essential medicines are available free of charge in the public sector, unaffordable transport costs were identified as a cross-cutting theme. In some instances patients could not afford to pay for transport to the nearest facility because of poverty. However, many other times, their circumstances were exacerbated by barriers identified under other dimensions of availability, accessibility, accommodation and acceptability. Differences in how medicines are distributed within various disease control programmes were viewed as an influence on the cost of accessing medicines i.e. ART availability in strictly accredited facilities poses a challenge for patients who have to travel longer distances because the nearest clinic does not offer ART. TB patients are among those who face the biggest transport challenges during their treatment period as they are required to make daily or weekly visits to the facility for their medicines. A TB nurse in East London estimated that some patients coming from afar pay approximately R60 (approximately seven United States dollars at the time of research) a day on transport and this was contributing to the defaulting rates.

### Empathic provider responses to address ATM barriers

The providers’ perspectives highlight the inadequacy of the existing formal responses in addressing many of the circumstances that impinge on equitable ATM. In confronting these challenges in their daily provision of care, providers adopt ad hoc decisions to meet the needs of their patients. There was agreement that because of patients’ low incomes and dependence on social grants, household savings are almost depleted before their next clinic appointment. Some providers attempted to align clinic appointments with social grant collection dates so that patients can collect their medicines and social grants on the same day. Others highlighted that this was helpful for the patients but it was impractical with large numbers of patients as it would create an imbalance in patient load and insufficient health personnel to deal with patients on the busier days.

In other cases, providers reported that they assessed the patient profile and provided what they termed ‘buffer’ stock for a few additional days (usually less than one week) in case patients come late for their appointment. In most cases, however, providers dispensed medicines for longer than the stipulated policy to patients they deemed stable, but cautioned them to return to the facility if they felt the need for medical attention before their next scheduled appointment. This was at the discretion of the prescriber, usually the nurse. There were variations in terms of how much stock patients received but between two weeks and a month were common for TB treatment that would otherwise be collected daily and two to three months for HIV and diabetes treatment which would otherwise be collected monthly. Although the decisions were made by prescribers, facility managers and pharmacy personnel were aware of this practice and supported it as highlighted below:

*“The sister in the chronic[care department] has now developed a system of giving the treatment to those that are stable…she must give them for three months …and then the fourth month they come for observations but those who are unreliable they must come monthly”.***(Facility manager, CHC4, rural)**

*“We do have those that are adhering so we give them their monthly supply. And because …they don’t want to use their (local) clinics, they come here and it is very far”.***(TB nurse, CHC2, rural)**

Occasionally, providers reported assisting desperate patients with their personal finances to enable them to return to their homes. An illustration that was given by a nurse in an urban facility:

“*…really we are stuck at times here with the patient, you will have to take your money, give patients to take taxis to their respective areas, because they are stuck here, maybe they are taken by the ambulance at night…there’s no money, there’s no escort, so you have to see to the finish. It is not easy to be a nurse, and when you look at us here, we are in an urban setting, think about the nurse who is working in the rural area; it is very much more difficult”.***(Chronic care nurse, CHC5, urban)**

### Provider views on future strategies to improve ATM

Providers described strategies that could be employed in the future to address poor quality of services in rural clinics, for example, having more sites accredited for ART or use of non-accredited sites merely as medicine collection sites and incentivizing doctors to work in rural sites so that staffing levels can be improved. One doctor indicated that she was the only doctor working at 27 clinics and this gave her an unreasonable work load. At the time of the research there was anticipation of a shift toward having all sites in the province managed by the Provincial Department of Health including those previously managed by the municipality. There was agreement that this move would improve coordination in the delivery of services and even resource sharing as indicated by one respondent:

*“…there was no cooperation between us and the municipality; it is going to be better now. Even if there is a shortage in a particular clinic, if I am here as a manager, I could allocate my nurses to that clinic”.***(Facility manager, CHC6, urban)**

We enquired about the potential role of mobile clinics in improving distribution of medicines although none of our tracer medicines had been distributed through the mobile clinics. Providers felt that mobile services have been geared toward dealing with minor ailments and as a result patients with comorbidities collect part of their medication from the mobile clinic and additionally, have to travel to the facility to collect their chronic medicines. This results in patients abandoning the mobile clinic completely and going to the facility where they can collect all their medicines simultaneously.

At an operational level, mobile clinics have been plagued by many challenges such as unavailability of vehicles committed to this service, bad roads and weather conditions which hinder physical accessibility and shortages of staff at the facility level which results in community appointments being cancelled as highlighted by these respondents:

*“Our staff shortages mainly affect the mobile services where we only have two professional nurses and one nursing assistant. According to the organogram, we are supposed to have two teams with five professional nurses”.* (**Facility manager, CHC2, rural**)

*“…at times they (service providers) have transport challenges so they will be disappointing those patients. They used to wait a long time, so they decided not to wait for the mobile clinic, they decided to come and get their treatment here. So if the mobile is not coming if that appointment date is cut, they wait there because of the transport problems”. In addition, “…sometimes they (patients) used to say we are not getting all the treatment, others are out of stock, so they do come (to the CHC) for other treatment”.***(Chronic care nurse, CHC3, rural)**

Issues pertaining to resources might be easier to address but addressing the needs of patients with comorbidities remains a challenge in areas where mobile clinics are not a supplementary service to the local clinic but are the only available means of service nearest to the people. While it is acknowledged that mobile clinics have the potential to address access barriers in hard to reach areas, some providers were sceptical about providing medicines such as ART through the mobile clinic citing intense administrative procedures associated with ART programmes, potential lack of privacy and limited contact time between patients and providers as the mobile vans move to different locations.

## Discussion

Our findings emphasize the role of providers as healthcare brokers with respect to their creative responses in addressing patient needs and mitigating health system shortfall. A key benefit of this study was the ability to draw experiences across different essential medicines in both urban and rural settings and with providers who have different roles in the care pathway. The reported perspectives were largely shared across providers of different essential medicines. Our key informant interviews highlight important bottlenecks and system failures that correspond to the multiple dimensions of access [7] and the practical dilemmas that providers face in dealing with them. Providers are directly impacted by these challenges on the frontline of care and this study highlights the proactive strategies that they employ to ensure that patients obtain and remain on treatment. How providers enact the role of broker between the system and their patients is critical to consider in terms of the broader problem of medicine accessibility and health services in general. There is a need for broad-based changes encompassing all disease areas to improve ATM. In doing so, however, there is a need to take into account three tiers of brokerage: (i) ATM barriers with no solutions offered yet (e.g. bottlenecks in the medicines supply chain and socio-economic and cultural factors), (ii) ATM barriers with potential solutions outside the health providers’ power (e.g. building improved transport networks) and (iii) ATM barriers with solutions already being implemented (e.g. flexibilities in prescribing and dispensing to suit patient needs). Implications for practice are discussed through presentation of a number of mechanisms with a set of critical questions to ask when addressing ATM.

First, the medicines supply chain is undermined by severe resource constraints and structural weaknesses often leading to stock outs and provision of inferior services. Patients respond to system barriers by ‘shopping’ for better services within the system or with alternative providers if they can afford the associated costs. Otherwise, they give up [12]. Similar to another study in South Africa [22], providers highlighted haphazard movement of patients within the health system as one of the threats to the down referral program. This emerged as a key factor that prohibits efficient planning related to medicines supply at primary level facilities. The supply chain needs to be equipped to deal with the growing demand for consistent supply of medicines, especially chronic medicines and this involves proper planning at all levels.

Second, context and patient characteristics play an important role in provision of medicines. For example, circular migration emerged as a key feature of the patient population yet health services tend to assume that patients are permanently situated in a particular locus. As a result, many patients are lost to follow up or default on treatment when they temporarily migrate from one place to the other. Also, relative to urban patients, rural patients face more access barriers as a result of poverty and underdeveloped transport infrastructure. Peters and colleagues found that the poor in low and middle income countries (LMICs) are consistently at a disadvantage in each of the dimensions of access and their determinants [23]. Another South African study also highlighted the financial burden of costs associated with health care on patients [8]. Both supply- and demand-side barriers carry a financial consequence for patients, particularly with respect to transportation. For example, patients incur costs for greater distances travelled in dealing with underdeveloped transport infrastructure or medicine stock outs in more proximate clinics. Patients’ ability to afford health related costs depend, among other things on any earnings, the composition of the social grants that a household receives, the size of the household and the household priorities. In light of this, improving access to medicines requires engaging in intersectoral actions between health, welfare and infrastructural development. Organization of “circular” public transport, instead of only “centripetal” transport, which gives users incentives to travel to town, instead of to the local health centre, is required.

### Implications for policy and practice

Findings from this study suggest that many providers are aware of the existing barriers to accessing medicines. They are taking advantage of the flexibilities within the health system, which enables them to make critical, responsible and responsive decisions in practice. They are attempting to address these challenges in creative and empathic ways. Indeed, clinical empathy is a quality that is deemed critical in determining patients’ ability to cope with both illness and quality of care and to improve health outcomes [24,25]. Our findings allude to the commitment of providers and the important role that they are playing in discovering the needs of the populations they serve and the efforts to address them. This draws attention to the social reality of relationships and interactions occurring at the patient and provider level and offers a different view to previous studies which found this relationship to be largely negative [12,26]. It is plausible that continuous patient-provider interaction, as evidenced by the average of eight years that health providers work in the same facility, promotes nurturing of positive relationships wherein providers better understand the challenges that patients face.

However, the practice of prescribing and dispensing for periods beyond what is lawfully allowed deserves careful attention, given the consequent impact of less frequent monitoring and patient follow-up. Providers however, suggest that dispensing medicines to stable patients with chronic conditions for up to six months would help reduce travel costs and de-congest health centres. They do not view this strategy as a threat to public health because patients are free to return to the facility as needed. Although prescribers have devised ways of evaluating patient stability through assessing health outcomes over time, the standard differs from place to place. The current variation in dispensing practices in order to address patient concerns raises important questions for policy and suggests a need to look into whether there is merit in institutionalising the informal strategies currently employed from an evidence-based point of view. Recent studies report that with the right kind of community support, it is feasible to provide treatment for longer periods [11]. Also, a recent program in South Africa has piloted provision of ART for longer periods with good results [27]. In the absence of standardised and evidence-based dispensing practices in order to address patient monitoring, the informal strategies reported in this paper may not produce optimum patient therapeutic outcomes.

It is also worth noting, that the current strategies employed by providers favour patients who are deemed stable and might further discriminate against patients who are unable to visit health centres because of multiple barriers. Diversification of strategies to improve ATM is absolutely critical. Diversification involves looking beyond the conventional facility-based approach by considering, among others, community based models which maintain identifiable links with the health system to increase the trust of the users.

Finally, the emergence of chronic non-communicable diseases and multiple morbidities is fast becoming the rule rather than the exception and efforts to pre-engineer the system towards a comprehensive, integrated, horizontal primary health care system should be considered. For instance, a recent analysis of mortality causes in South Africa has highlighted more deaths resulting from non-communicable diseases such as diabetes than from HIV [28]. A responsive system takes into account the limitations of a vertical system while at the same time ensuring that medicines for priority conditions are not compromised [29]. Responsiveness also requires taking into account characteristics of the population served to ensure that circular migrants have continuous ATM and to address other social, cultural and economic barriers more appropriately. Ultimately, addressing these ATM barriers creates a healthier working environment for providers and protects them from situations where, for example, they occasionally feel obligated to assist stranded patients.

## Conclusion

Effective ATM to minimise treatment interruption requires addressing supply- and demand- barriers concurrently with a consideration for context. The evidence presented in this paper shows the importance of all five access barriers (availability, accessibility, accommodation, acceptability and affordability) and the complex ways in which they interact with each other. Issues that require further research include diversifying methods for distributing medicines in rural settings, understanding whether the brokerage mechanisms adopted by providers actually improve ATM, and determining whether such practices are appropriate, feasible and sustainable. It is also critical to understand the characteristics of the population served to ensure that circular migrants have continuous ATM. Finally, the brokerage role of providers is underutilised and we recommend that as ongoing practice, they should be actively engaged in intersectoral discussions aimed at improving the health care response.

## Abbreviations

CHC: Community Health Centre
AMASA: Accessing Medicines in Africa and South Asia
TB: Tuberculosis
ART: Anti-retroviral treatment
KZN: Kwa-Zulu Natal
WHO: World Health Organisation
LMICs: Low-and-middle income countries
